# Arginine deprivation, growth inhibition and tumour cell death: 2. Enzymatic degradation of arginine in normal and malignant cell cultures

**DOI:** 10.1038/sj.bjc.6600681

**Published:** 2003-02-18

**Authors:** R Philip, E Campbell, D N Wheatley

**Affiliations:** 1 Department of Cell Pathology, University of Aberdeen, MacRobert Building, 581 King Street, Aberdeen AB24 5UA, UK

**Keywords:** arginase, arginine decarboxylase, arginine, catabolism, deprivation, proliferation, cell death, citrulline, ornithine, agmatine, HeLa, L1210, cancer therapy

## Abstract

*Arginase* added to culture medium reduced arginine to negligible levels within ∼6 h, and enzyme activity persisted relatively undiminished for at least 3 days. Human and bovine arginase proved equally effective. The response of normal cells was to enter G1 (G0) arrest, from which most of the cells could be recovered weeks later. In contrast, malignant cell lines treated with unpegylated or pegylated enzyme resulted in cell death on a massive scale within 3 – 5 days, with a very low to negligible percentage of cells (<0.01%) being recoverable on restoration with arginine. Although pegylation resulted in a 40% drop in specific activity, arginase was considerably more stable and remained active for ≫8 days. *Arginine decarboxylase* caused malignant cell arrest at the same units per millilitre as arginase. Its breakdown product, agmatine, was relatively nontoxic in the presence of arginine, but exacerbated cell death above millimolar concentration in its absence. Although ornithine failed to rescue cells from deprivation, citrulline recovered cells in all cases, although less well in fast-growing tumour cell populations, whereas readdition of arginine failed to work unless a complete medium change was given (because of the persistence of the enzymes in the medium catabolising its destruction). The advantages and disadvantages of these two arginine-catabolising enzymes are discussed, and compared with *arginine deiminase*.

A major aim of our work has been to lower arginine levels *in vitro* and *in vivo* to arrest the growth of cells, and selectively cause the death of cells of malignant phenotype (Scott *et al*, 2000; Wheatley, 1998). Normal cells with stringent checkpoints respond to nutrient deprivation by arresting cells in G0, from which the majority can be recovered several weeks later. Lowering arginine levels presents no difficulty in culture, and most previous studies have concentrated on arginine-deficient media containing dialysed serum. A closer comparison with *in vivo* work would be to degrade arginine *in situ* with enzymes, the three main contenders being arginase, arginine decarboxylase and arginine deminase. The ultimate objective is to sustain free plasma or medium arginine at <5 *μ*M (preferably ∼1 *μ*M) long enough to procure cell death, which is clearly going to be much more difficult to accomplish *in vivo* than in culture. Our present experiments were designed to give further insight into the comparative efficacy of two of the arginine-catabolising enzymes *in vitro*, viz arginase and arginine decarboxylase, which could lead to improvements in their deployment in the *in vivo* treatment of cancer. Arginine deiminase will only be discussed here, because it generates ammonia and its toxic action on cells presents a problem that makes it less useful as a potential therapeutic agent (Wheatley and Campbell, 2002). The important enzymes of arginine metabolism relevant in the present studies and their main properties are given in Table 1

**Table 1:** Properties of some of the more relevant arginine-metabolising enzymes in this study

**Enzyme**	**EC number**	**Substrate(s)**	**Cofactors/ions/etc**	**Products**	***K***_**m**_ **(mM)**
Arginase	3.5.3.1	Arginine	Mn^2+^	Ornithine and urea	10.5
Arginine decarboxylase	4.1.1.19	Arginine	Pyridoxal phosphate	Agmatine and CO_2_	∼1
Arginine deiminase	3.5.3.6	Arginine	Mg^2−^	Citrulline and NH_3_	∼0.1–0.2
Nitric oxide synthase	1.14.13.39	Arginine (made from citrulline) and O_2_	NADPH→NADP+	NO and citrulline	0.03

.

The specific objectives in the experiments reported here were to demonstrate that (i) arginase will quickly and effectively mop up all free arginine in culture medium for a considerable time measured in days after a single treatment, (ii) citrulline in the absence of arginine will generate arginine, which is *not* accessible to degrading enzymes in the medium (i.e. the ‘free’ arginine does not equilibrate with the medium), and (iii) different cell types clearly have different citrulline requirements, which correlates with growth rate. Thus, the rate of conversion of citrulline to arginine becomes the rate-limiting step, and this becomes most obvious the faster the cells grow.

Removal of arginine from the body under normal physiological conditions results in a powerful boost to the homeostatic mechanisms by which free amino-acid levels in the blood are restored. In addition to the release of considerable amounts of free amino acids liberated by skeletal muscle breakdown, the gut/kidney axis (Wu and Morris, 1998) is involved in arginine metabolism, since citrulline biosynthesis occurs in the former organ and is converted to arginine in the urea cycle by the actions of argininosuccinate synthetase (AS; EC 6.3.4.5) and argininosuccinate lyase (AL; EC 4.3.2.1) in the kidney (Schimke, 1964).

Some cells are clearly more adept than others at circumventing arginine deficiency by recycling enzyme products. The conversion of ornithine to citrulline requires ornithine carbamoyl transferase (OCT; EC 2.1.3.3), but apart from normal hepatocytes and a few minimal deviation hepatomas, most cultured cells cannot perform this transfer (Sugimura *et al*, 1992), and therefore ornithine rarely substitutes for arginine in culture (Eagle, 1955). On the other hand, Eagle (1959) found that citrulline can substitute for arginine, and this is so in all malignant and normal cells cultured in aeginine-free medium (AFM) that we have so far investigated (Wheatley *et al*, 2000). The question now is whether citrulline metabolism interferes with or circumvents enzymatic efforts to reduce free arginine to negligible levels in cultures.

Arginine has arguably the most complex metabolic pathways of any amino acid within the cell and the body. It can be removed by dietary restriction, dialysis or enzymatic destruction. The first method has always presented problems in the preparation of suitable diets, and of compliance (Wheatley, 1998). The second is experimental, and the technology needed to achieve low blood arginine is highly complicated (Wheatley *et al*, 2002), although it is being further developed (Shettigar, 1990). The third approach has been adopted by a number of groups, and involves enzymatic degradation of arginine *in situ* (Bach and Swaine, 1965; Storr and Burton, 1974; Terayama *et al*, 1982; Gong *et al*, 2000; Miyazaki *et al*, 1990). In all probability, the combined use of these strategies will be required.

An adequate supply of arginine is vital for growth, and its removal in early life seriously stunts normal development (Daniel and Krishnan, 1967). This might later be reflected by limited tumour growth in adult animals, where supply again falls short of demand; hence, the danger is that arginine supplementation enhances tumour growth and makes matters worse (Bach and Lasnitski, 1947; Leffert and Paul, 1973). The effects of arginine withdrawal become manifest more quickly than that of any other amino-acid withdrawal, with the additional advantage that tumour cell death occurs quickly without further intervention (Wheatley *et al*, 2000). For tumour regression to occur, arginine probably needs to be down at micromolar levels for 7 – 8 days, although lymphomas and leukaemias may succumb in 2 – 4 days (Storr and Burton, 1974; Takaku *et al*, 1992). Although we have already reported on the effects of AFM with 25 cell lines (Scott *et al*, 2000), comparative studies on the efficacy of arginine-catabolising enzymes added to normal medium (enzyme-treated medium, or ETM) were undertaken as an alternative means of achieving a deficient state.

## MATERIALS AND METHODS

### Cell culture

L1210 cells (murine lymphoblastic leukaemia) were maintained in flasks at between 0.1 and 3×10^6^ cells ml^−1^ in exponential phase growth (generation time ∼15 h) in RPMI 1640 medium (Life Technologies, Paisley, UK) at 37°C in a humidified atmosphere of 5% CO_2_. For experiments, they were set out in 2 ml of culture medium at 5×10^4^ cells ml^−1^ in 12-well plates. HeLa cells (cervical carcinoma) and human diploid fibroblasts (the latter being early passage numbers from primary cultures grown from human skin taken at breast reduction) were seeded at 50 000 ml^−1^ in flasks or plates in a similar manner previously described in Scott *et al* (2000). The cells were used at the lowest passage numbers that would provide adequate stocks of cells, they were checked as mycoplasm free, and they were authenticated from source, as required by the UKCCCR ‘*Guidelines on the use of cell lines in cancer research*’ (<http://ukcccr.icnet.uk>).

Basic RPMI 1640 medium (Sigma, Poole, Dorset, UK) lacking the following supplements was prepared by the addition of these compounds to the stated final concentrations: 4 mM L-glutamine, 0.4 mM L-arginine, 0.2 mM L-cystine, 11.1 mM D-glucose, 0.2 mM L-inositol, 0.38 mM L-leucine, 0.1 mM L-methionine. Foetal calf serum (10%) (Harlan, Crawley Down, UK) was added for stock growth, but 5% dialysed serum was used in experimental plates. AFM omitted L-arginine from the final medium formulation. Normal (positive) controls were given AFM to which arginine had been restored at 0.4 mM, ensuring that the same medium was used throughout, with negative controls given placebo (vehicle) only, and cultures treated with other amino acids received these *in lieu* of arginine. Cells were counted electronically with a Coulter Counter at regular (usually daily) intervals. [Note: RPMI 1640 normally has an arginine concentration of 1.0 or 1.1 mM. However, in order to compare with most of our previous studies where arginine was kept at 0.4 mM (see Scott *et al*, 2000), this same (more critical) level was used in the present studies.)

### Enzyme preparations (for additional data, see Table 2)

#### Arginase

**Table 2:** Details of relevant and associated enzyme preparations

**Enzyme preparation**	**Source**	**Species**	***K***_**m**_ **(mM)**	**Specific activity U**
Arginase				
	Sigma	Bovine (articular cartilage)	5–10	192
	Ikemoto	Human (recombinant)	5–15	1482
	Ikemoto; pegylated	Human (recombinant)	ND	595
Arginine decarboxylase				
	Sigma	*E. coli*	∼1	5–15
	Boyle	Human (recombinant)	∼1	2.5
	Boyle; pegylated	Human (recombinant)	Inactive	inactive
				
Extracts (arginase)	Liver	Bovine	5–10	0.5–3
	Liver	Human	5–10	1
				
Arginine deiminase (Gong *et al*, 2000)	See Holley (1967)	Human	∼0.1–0.2	
NO synthetase (Chang *et al*, 2001)	See Wheatley (1998)	Human	0.03	

Arginase – amount of enzyme required to convert 1*μ*mol arginine to ornithine and urea at pH 9.5 and 37°C. Arginine decarboxylase – amount of enzyme required to convert 1*μ*mol arginine to agmatine and CO_2_ at pH 5.2 and 37°C. Specific activity refers to arginase activity, and its level depends on extraction procedure, with preparations that have varied as much as 10-fold from sample to sample. Data for liver extracts, arginine deiminase and nitric oxide synthase are included only for information and comparison, because the affinities of the latter two can be very much higher (lower *K*_m_) than for the other enzymes.

Bovine liver arginase was obtained in its native form from Sigma with an activity of approximately 200 units mg^−1^ and was used in the experiments indicated without any further activation steps. Human recombinant arginase was obtained from Dr Ikemoto, Kyoto, Japan, with an activity of ∼1500 – 2000 units mg^−1^ protein. Prior to use, the enzyme was heat activated at 60°C for 10 min in the presence of Mn^2+^ ions, as instructed by Dr Ikemoto (personal communication). In order to prolong the half-life of the enzyme, it was also pegylated with cyanuric chloride-activated polyethylene glycol (Sigma) at room temperature, pH 9.0, for 30 min using the method of Savoca *et al* (1984). The resulting enzyme was dialysed against 5 l of 1×phosphate-buffered saline (PBS) to remove excess polyethylene glycol with a Hemoflow F40S capillary dialyser (Fresenius Medical Care, Bad Homburg, Germany). The recovered enzyme was filtered through a 0.2 *μ*m filter (Nalgene, Nalge Co., Rochester, NY, USA) and stored at −20°C ready for use.

#### Arginine decarboxylase

Sigma arginine decarboxylase from *E.coli* was used, which had a specific activity decidedly lower than arginase (see Table 2). However, human recombinant arginine decarboxylase expressed in *E.coli*, obtained frozen from Professor Steven M Boyle (Virginia Polytechnic Institute and State University, Blacksburg, VA, USA), had an even lower activity of ∼2.5 units mg^−1^. Owing to the latter preparation having a high protein content, the enzyme precipitated on thawing. Prior to use, the solution was warmed to 18°C and adjusted to 20 mM with respect to dithiothreitol (DTT). After incubating the solution on ice for 10 min, the preparation was centrifuged at 10 000 *g* for 10 min and the supernatant recovered. The supernatant was diluted 1 : 10 and dialysed against 0.9% saline along with the cofactors pyridoxal-5-phosphate (1.2 *μ*M) and MgCl_2_ (4 mM).

### Enzyme activity

Enzyme activities were analysed *in vitro* over time at 37°C in a humidified atmosphere of 5% CO_2_. *Arginase* was added to basic RPMI medium (without foetal calf serum) with L-arginine at 1.1 mM. The reaction was stopped at selected time points by the addition of 10% sulphosalicyclic acid (Sigma), which precipitated the protein at 4°C for 30 min. The resulting samples were centrifuged at 13 000 rpm for 10 min in a microcentrifuge and the supernatant was filtered through a Millex^R^-GV 0.22 *μ*m filter (Millipore, Bedford, MA, USA). Arginine and ornithine were measured using a Biochrome 20 Amino Acid Analyser (Amersham Pharmacia Biotech, Cambridge, UK). Arginase activity was calculated in units, 1 unit being the amount of enzyme catabolising 1 *μ*mol of arginine to ornithine (and urea) per minute per milligram of protein. Similarly, a unit of arginine decarboxylase was the amount of enzyme catabolising 1 *μ*mol of arginine to agmatine per minute per milligram of protein.

### Ornithine assay

Arginase activity was also assayed by the method of Ochoa *et al* (2000), which uses ninhydrin to detect the ornithine concentration, within each sample by a colorimetric procedure measured on the spectrophotometer at ∼515 nm wavelength (Chinard, 1952). The specific activity of arginase can therefore be defined as the amount of ornithine (*μ*mol) per minute per milligram of protein produced, calibrated against known standards within the range 10–100 *μ*M.

### Protein analysis

An adaptation of the Lowry method (Oyama and Eagle, 1952) was used in conjunction with the above ornithine assay to determine accurately the protein concentrations of the various samples. The specific activities of the enzymes were required for accurate comparisons between the different preparations and batches.

### Statistics

Comparisons have been analysed by the Student's ‘*t*’-test only where appropriate, with significance being taken as *P*<0.05. Small deviations have not been subject to rigorous inspection at this stage because these will not provide the level of discrimination sought between malignant and normal cell responses.

## RESULTS

### Human diploid fibroblasts

**Figure 1:**
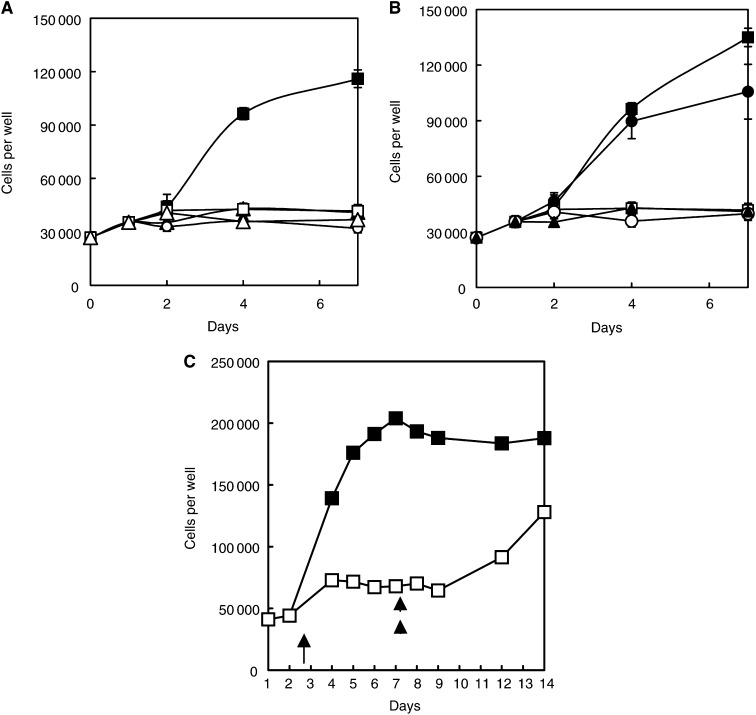
(**A**) Growth kinetics of human diploid fibroblasts in (▪) arginine-containing medium (positive controls), (□) AFM, and (○) arginine-containing medium plus 1 unit ml^−1^ arginase, or (▴) the same except with arginine decarboxylase at 1 unit ml^−1^, starting at day 1 (the latter was controlled with its own vehicle without arginine present in the curve with the ▵ symbols). The total number of cells in each of three wells was averaged at each time-point, and the bars given are 1 s.d. around the mean. The significance of the differences between (▪) and (○) or (▴) is very highly significant (*P*<0.001), but the differences between (□), (▵) and (○) are not significant. (**B**) Growth kinetics as in (**A**), but for cells with attempted rescue in the continued presence of the enzyme of the arginase-treated fibroblast cultures with 0.4 mM arginine (○), 0.4 mM citrulline (•) or 0.4 mM ornithine (▴). The difference between the positive control and the citrulline rescued cells was not significant (*P≫*0.05). (**C**) Recovery of fibroblast from 5 days of arginine deprivation prior to the addition of 0.4 mM arginine to the culture medium without a medium change. (▪) Positive controls that have had AFM but arginine restored immediately after a medium change at day 1, compared with a second group (□) in which arginine was restored only on day 6, that is, after 5 days of complete arginine deprivation. Cell loss during this whole experiment was minimal.

Fibroblasts growing with a generation time of about 36 h were arrested in AFM. The cell number remained almost constant for >6 days (Figure 1A

), although sometimes a small initial fall in number was seen. The cells could be kept at 37°C under these deprivation conditions for at least 3 weeks and probably considerably longer without serious loss of viability, although we show in Figure 1B rescue following a medium change into arginine-containing medium after 5 days of deprivation. There was little cell loss early in the rescue, but recovery required 5 – 6 days before growth kinetics approached those of the positive controls (which by this time had reached stationary phase).

Almost identical data were recorded for cultures in complete medium treated with either bovine arginase or *E. coli* arginine decarboxylase (hereafter ETM1 and ETM2, respectively) *in situ* (Figure 1A). Arrested cells entered G1/G0, as previously described in AFM by Scott *et al* (2000). In terms of cell growth inhibition kinetics sampled after 3 days treatment, arginase was active at 0.25 – 0.5 units ml^−1^ (IC_50_ ∼0.3 units ml^−1^), the inhibition becoming increasingly felt with time. It was more immediately and completely inhibitory at 1 unit ml^−1^ and above, but with higher enzyme concentrations of up to 5 units ml^−1^, depression of cell growth occurred earlier. The same was true of arginine decarboxylase (Figure 1A), but dose – response curves will be dealt with in regard to the malignant cell lines for L1210 cells below. However, these results refer here to slow-growing fibroblasts, and we already know (Wheatley *et al*, 2002) that 1 unit ml^−1^ arginase can reduce arginine to negligible levels in a matter of a ∼3 – 6 h *in vitro* (see below and Discussion).

Citrulline added to the ETM1-treated cultures without removal of the enzyme permitted growth at approximately control rates (*P*≫0.05), and at the same rate in AFM cultures (only one curve shown in Figure 1B for the sake of clarity). It was noteworthy that arginine failed to restore cell growth in ETM1-treated cultures.

Data for ornithine ‘rescue’ have been included in Figure 1A, but will be omitted throughout the rest of these studies because it (a) allowed no recovery under any of the deprivation conditions we have used (in agreement with earlier findings of Wheatley *et al*, 2000), and (b) lacked any observable inhibition of ETM1, on which it is supposed to act by feedback inhibition (see Discussion).

ETM2 had a similar effect at 1 unit ml^−1^ to arginase (Figure 1A), and also responded to citrulline in an almost identical manner (Figure 1B, curve not shown for the sake of clarity). Arginine once again failed to allow recovery of ETM2-treated cultures in the continued presence of the enzyme, although the recovery of AFM-treated cells was identical to the positive control. With regard to the latter, recovery after a 5-day treatment with AFM required >3 days to re-establish normal growth kinetics in fibroblasts (Figure 1C).

### HeLa cells

**Figure 2:**
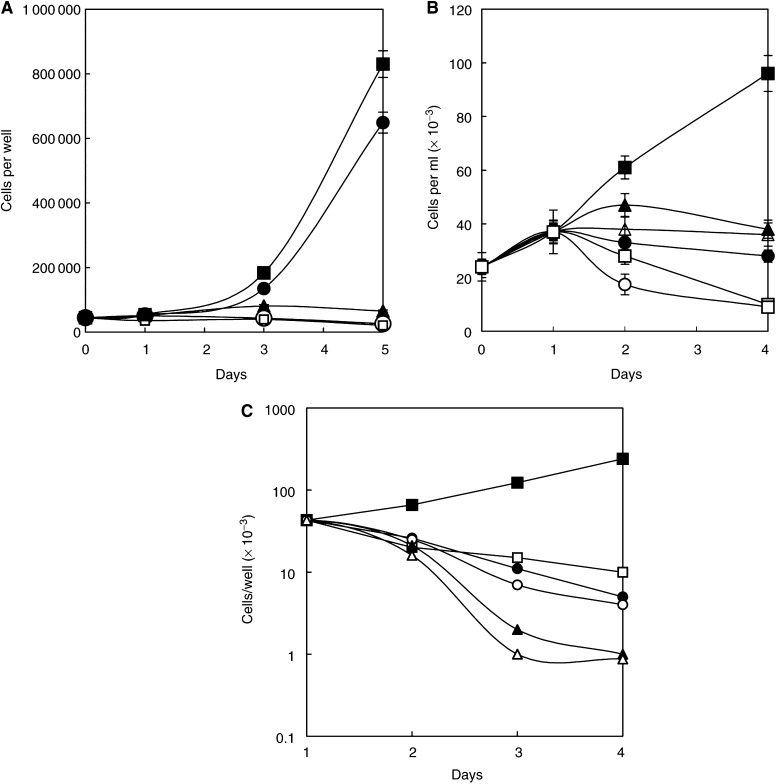
(**A**) HeLa cells treated with arginase (○) at 1 unit ml^−1^ compared with cells in AFM (□) or its positive control with arginine present at 0.4 mM (▪), as in Figure 1A. Neither arginine (▴) nor ornithine (curve not shown) rescued the cells treated with arginase, but citrulline did (•), with the difference between the open and the closed circle being very highly significant, and the difference between (▪) and (•) just significant (*P*<0.05 but >0.01). Details of the curves as above, with error bars of 1 s.d. around each mean value sometimes not appearing outside the symbol. (**B**) Effect of arginine decarboxylase on HeLa cells growth in normal medium (▪) or AFM (□). The dose–response effect of the enzyme can be seen after being added at day 1, showing that the initial stages between day 1 and 2 gave a wide range of growth inhibition, see inset series labels. ADC was added at the following units per ml to normal medium: ▴ 0.1; ▵ 0.5; • 1; ○ 5. It is noteworthy that the highest dose level (○) of 5 units ml^−1^ was quickly and markedly effective (see Results for probabilities, and Discussion for comments on dose–response relations). (**C**) Effects of agmatine–the product of arginine decarboxylase–on HeLa cells, as tested in AFM, shown on a semi-log plot. The positive AFM control curve is (▪), whereas the AFM (negative) control (□) proved the least affected of the deprived cells, but concentrations of >1 mM agmatine in the absence of arginine were required to produce ‘significant’ damage, that is, 4 (▴) and 10 mM (▵), respectively. Less effective were 0.4 mM (•) and 1.0 mM (○). The data are averages of means, s.d. bars cannot be given and therefore ‘significance’ has no statistical validity, but a clear dose-dependent trend is seen.

**Figure 3:**
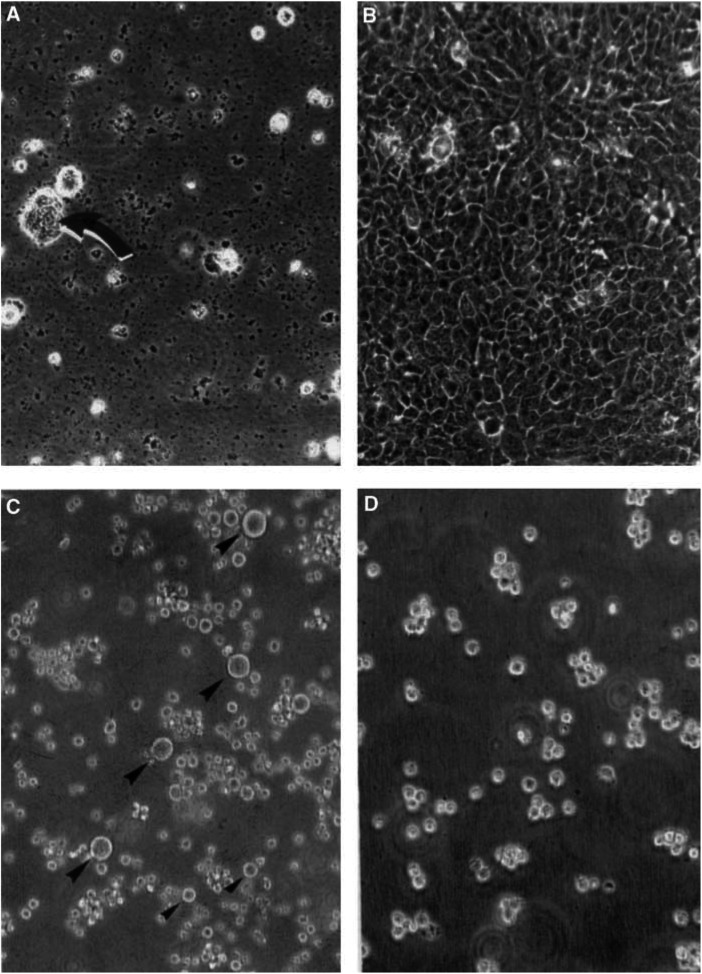
(**A**) HeLa cells subject to arginase treatment for 5 days; virtually no intact cells remain present except for a small clump (arrow) in which a large multinucleate cell is present. (**B**) Control HeLa cell culture in which no arginase was present for 5 days and has gone to confluence. (**C**) L1210 cells in a settled suspension from a culture after 3 days of arginase treatment; the majority of particles are shrivelled cells or free nuclei, with a few intact cells still present (small arrowheads), but several large, intact multinucleate (polyploid) cells are present in this particular field (large arrowheads). Nuclei remain in culture when L1210 cells break down because of the paucity of lysosomal enzymes. (**D**) Control L1210 cells, seen in the plane of focus in suspension. The images are all phase-contrast micrographs of living cultures. Magnifications are approximately ×200 for all panels.

The behaviour of HeLa cells (generation time 23 – 24 h) to the presence of ETM1 and ETM2 was also very similar to our previous findings using only AFM, and are included in the current experiments for comparative purposes (Figure 2A

). After the second day, cell death became progressively more obvious and by 5 days, few, if any, cells remained intact except for some large multinucleated cells (Figure 3A and B

). The effective dose – response curve was similar to that of fibroblasts, and citrulline circumvented the effects of AFM and ETM1, but here a significantly reduced growth rate of HeLa cells given 0.4 mM citrulline was observed, that is a level corresponding to the 0.4 mM arginine used throughout this work (Figure 2A, *P*<0.05>0.01). Once again, adding arginine to cultures treated with enzymes failed to rescue growth because, unlike citrulline, it was quickly degraded.

The response of HeLa to arginine decarboxylase was very similar to that seen in fibroblast cultures, with good inhibition seen at 1 unit ml^−1^, but more immediate and obvious cell death occurred within 4 days with 5 units ml^−1^ (Figure 2B), although in terms of cell growth arrest measured after 3 days, the dose-response curve for ETM2 looks particularly sharp (see data below for L1210 cells). It was noteworthy, however, that 0.1 units ml^−1^ brought about cell arrest and the start of cell death after 2 days (*P*<0.1) and a highly significant fall by day 4 (*P*≪0.01), indicating persistence of the enzyme as it gradually got to work with time (this enzyme being of higher affinity but lower capacity than arginase). Recovery with citrulline was effective, whereas arginine was again ineffective (the data mimic that of arginase recovery and are therefore not shown here).

To determine whether agmatine, the product of arginine decarboxylase, might *per se* have some effect on cell growth, we analysed it by adding agmatine as an exogenous preparation in the presence and absence of arginine. In the presence of 0.4 mM arginine, agmatine had no obvious effect, except some cytotoxicity at 5 mM. In the *absence* of arginine, agmatine was decidedly cytotoxic, progressively reducing HeLa cell number to well below that of the negative controls (*P*<0.01) as the dose increased and most notably at and above millimolar concentrations (Figure 2C; see Discussion).

### L1210 cells

**Figure 4:**
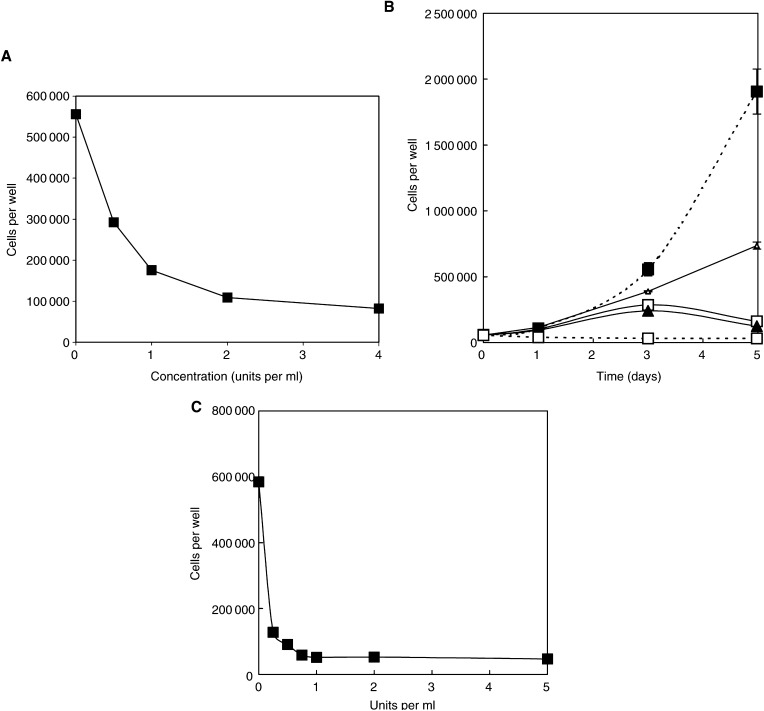
(**A**) Dose–response curve showing the inhibition of L1210 cell growth by Sigma (bovine liver) arginase used in the experiment shown in Figure 9. This preparation had about half its original activity after storage for some months, but shows that at a putative strength of 1 unit ml^−1^ (approximately equal to 0.5 units ml^−1^ of the original enzyme), it slowed cells after 3 days when cell counts were made, but not as immediately as a higher dose halted growth. The lowest values towards the higher enzyme concentrations correspond to the original inoculum of L1210 cells per well. (**B**) Effect of 0.5 units ml^−1^ arginase (Sigma) on L1210 cells in suspension in arginine-containing medium given at day 1 (▴). The positive and negative controls in AFM are given as before by the symbols (▪) and (□), respectively, but with dashed lines (*P*≪0.01). They show the expected responses. Enzyme-treated cultures (solid lines) and arginine (▴) follow the same curve as the arginase alone (□), but the arginase-treated culture given 0.4 mM citrulline (▵) shows recovery. However, its growth is ∼30% the rate of the positive control, the difference being highly significantly slower (*P*≪0.01). The symbols of the last curve have been reduced to enable the 1 s.d. bars around the means of triplcate values to be seen. (**C**) Effect of arginine decarboxylase on L1210 cells in terms of the action of a Sigma preparation. The enzyme was freshly prepared and highly active at 1 unit ml^−1^ within the 3 days at which assessment was made (cf. Figure 6A). The lowest value given here at the high concentrations corresponds approximately to the original inoculum density of the cells in each well (ie 100 000).

A series of similar experiments were carried out with L1210 cells, which have an exceedingly rapid growth rate (generation time 15 h) and often attain a final concentration of over 3 million cells per millilitre in suspension in normal medium. Clearly, this cell line has a considerably higher nutrient demand than HeLa cells, and a far greater one than fibroblasts. The basic findings confirm the above HeLa cell data, but the faster utilisation of arginine meant that cell death was more rapid in its absence, whether by AFM or ETM treatment (Figures 4A and B

). Probability estimates are given in the figure legends.

The dose–response curve for arginine decarboxylase (Figure 4C) shows its considerable efficacy against L1210 cells after 3 days treatment. Most cells were irrecoverable after 4 days of AFM or ETM treatment. A few cells persisted, as shown by microscopic examination, these being predominantly large multinucleated cells, as seen with HeLa cell cultures (see Figure 3C and D, and Discussion).

**Figure 5:**
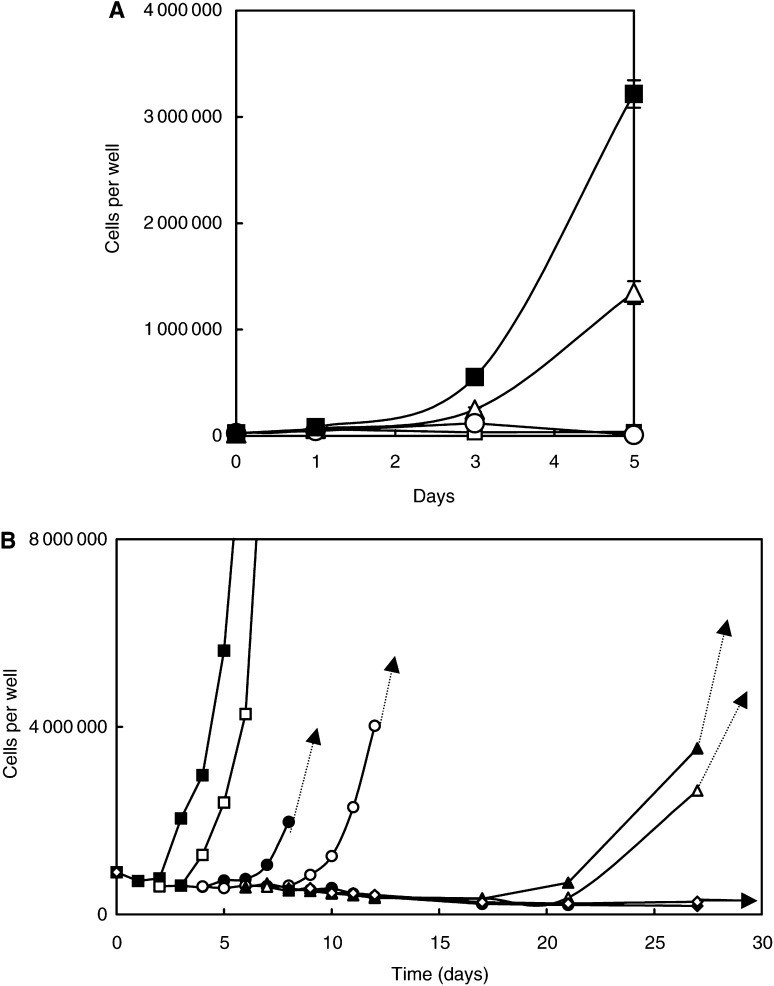
(**A**) Effect of human recombinant arginine decarboxylase on L1210 cells. The data show the pronounced inhibition of growth of L1210 cells in the presence of this enzyme at 1 unit ml^−1^ (○), which is the same as in the negative control cells in AFM (□). Note that the 0.4 mM citrulline rescue (▵) gives only ∼40% of the growth rate of the positive control (▪), the difference being highly significant from positive controls lacking the enzyme (*P≪*0.01), but also equally significant in extent against the unrescued control. (**B**) L1210 cells were treated with 1 unit ml^−1^ human recombinant arginase for periods up to 27 days, but rescued by medium change into complete RPMI 1640 with 10% normal foetal calf serum at 1 (▪), 2 (□), 3 (•), 4 (○), 6 (▴), 7 (▵), 8 (♦) and 9 (⋄) days. Recovery in the last two cases finally occurred at around 40 days (not shown), but the kinetics were not followed by cell counts.

Citrulline rescued the cells under AFM and ETM1 (arginase) or ETM2 (arginine decarboxylase) treatment, but arginine could only rescue cells from AFM (Figure 4A and Figure 5A

). Of interest is the finding that 0.4 mM arginine sustained normal growth when returned to AFM medium shortly after the start of incubation, but the kinetics of recovery were very significantly reduced with 0.4 mM citrulline (Figure 5A; cf HeLa cells in Figure 2A).

### Recovery by removal of enzyme and arginine restoration

In the continuing presence of arginase, only large cells remained intact in L1210 cultures (Figure 3C and D), from which repopulation of the cultures on removal of the enzyme presumably occurred (see Discussion). Among L1210 cells that had been allowed to die for as long as 4 days in AFM, some began to show signs of recovery between 3 and 6 days later in arginine-containing medium, but cells left longer in AFM usually took 2 – 4 weeks to start growing exponentially again (Figure 5A). This experiment went for 27 days, by which time the cultures rescued after 8 and 9 days had not recovered. However, around 40 days later these cultures also rapidly grow. In subsequent experiments where cells were deprived for 14 days, a small percentage of wells (an average of ∼2 – 3 per 12-well plate) showed that growth had resumed between 3 and 4 weeks after rescue (see Discussion).

### Recombinant human arginase and ADC

**Figure 6:**
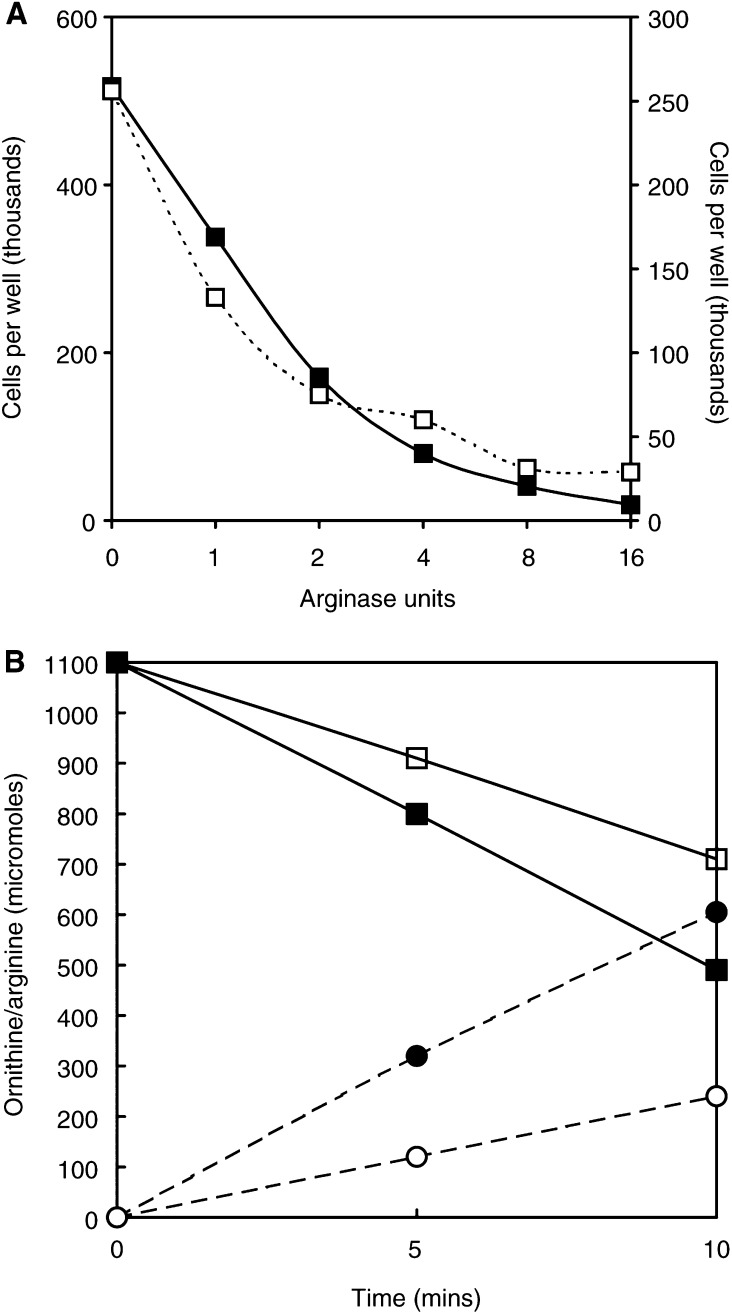
(**A**) Human recominant arginase prepared by Ikemoto *et al* (1990) was divided into two batches and compared with regard to inhibition of L1210 cells over two days, showing a dose-dependent effect similar to Figure 8 for HeLa cells. One of these batches was pegylated and compared with the other in terms of the breakdown of arginine to ornithine in the next figure. (**B**) Alterations in the levels of arginine and ornithine in RPMI medium measured in the amino-acid analyser after incubation for 5 and 10 min, to show their relative rates of arginine loss in unpegylated (▪) and (□) pegylated enzyme, and correspondingly the rise in ornithine in unpegylated (•) and pegylated (○) arginase treatment set at precisely 1 unit ml^−1^ of the original preparation in both cases.

The two preparations of recombinant human arginase expressed in *E. coli* had considerably higher specific activities than commercially available preparations of non-human enzymes (Table 2; Ikemoto *et al*, 1990). However, growth kinetics were affected in both HeLa and L1210 cell lines in a manner similar to any of the enzymes used in this study when they were given at equivalent units of activity (Figure 6A

), all of which were fully effective for >3 days at 37°C. Pegylation of arginase reduces its activity (Savoca *et al*, 1984), and our pegylation produced a 60% fall in activity (Figure 6B); thus it took over twice as long to reduce arginine to below detectable levels in our assay (i.e. <0.5 nmol). Pegylated arginase was more stable than the unpegylated form, and persisted without diminished vigour in culture for at least 8 days and probably a lot longer. This is evidenced by the fact that adding arginine to pegylated arginase-treated L1210 cells and leaving them for periods of up to 2 weeks did not permit recovery when arginine was restored at 0.4 mM.

Recombinant ADC worked as effectively as the Sigma preparation, giving almost identical data at equivalent units per millilitre. In contrast to arginase, pegylation of recombinant arginine decarboxylase completely abolished its catabolic activity.

## DISCUSSION

*In vitro* work shows that arginine withdrawal can have a devastating effect on malignant cells relative to normal cells, irrespective of whether deprivation is achieved by straightforward manipulation of the medium or *in situ* enzymatic catabolism of arginine. This exercise is easy to carry out *in vitro*, but similar control over the availability of arginine *in vivo* will need to be gained. However, using enzymes as a treatment is going to be a more practical proposition than, for example, extensive dialysis of the blood (Takaku *et al*, 1993). Although the behaviour of a tumour in the body may not accord with their responses *in vitro* for many obvious reasons, we need to demonstrate that it does work as well in principle with enzymes as with deficient medium, already indicated in some previous reports (Storr and Burton, 1974; Terayama *et al*, 1982; Savoca *et al*, 1984; Miyazaki *et al*, 1990; Gong *et al*, 2000; Wheatley *et al*, 2002).

### Enzymes as potential therapeutic agents

The action of arginase is relatively swift and its capacity high (Figure 2B); it therefore clears the medium of arginine relatively quickly. Theoretically, the rate of the reaction at 1 unit ml^−1^ converts 1 *μ*mol arginine to ∼1 *μ*mol ornithine per minute (Figure 6A). In a medium or solution containing 400 *μ*M arginine, each millilitre contains 0.4 *μ*mol. In the presence of 1 unit of arginase, just 20 s should suffice to complete the conversion to ornithine. However, this calculation is based on arginase reacting with excess (4 mM) arginine under the conditions of a biochemical assay. The rate of conversion at low concentrations is slower, mainly because arginase has a high *K*_m_ (Table 2). In following the actual conversion rate *in vitro* with 40, 100, 400 and 1000 *μ*M arginine in PBS, it took about 20 – 50 min for the reaction to go to completion at 37°C. If many other amino acids are present, as in RPMI-1640, this time can be extended by a factor of 2 – 3, and even further when allowance is made for interference from 5% dialysed serum. Thus, it is in keeping with amino-acid analysis data that enzyme degradation takes at least an hour from the time of addition of enzyme to reach 10^−5^ M levels and between 3 and 6 h to be brought to negligible levels (10^−6^ M or less). We also know that HeLa cells do poorly when grown in medium containing 10^−5^ M *μ*M arginine (Wheatley *et al*, 2000), and therefore depression of growth should begin to be felt within an hour or two.

The product of arginase, ornithine, has the advantage that it cannot be reconverted *in vitro* to arginine by cells other than hepatocytes and a few minimal deviation hepatomas (Sugimura *et al*, 1992), which will happen in the body unless liver and kidney function are seriously compromised. In some respects this makes arginase less attractive in therapy, but it is still possible that the ornithine reconversion is too slow or quantitatively too small to maintain adequate arginine levels, and that the enzyme would still have some (restricted) action.

A disadvantage of ornithine could be its feedback inhibition of arginase (Schimke, 1964). However, no build-up of ornithine in cultures occurred to levels that noticeably reduced the efficacy of arginase. This is almost certainly because the enzyme was added exogenously and had not been produced and released *in situ*, as seen in the body following liver injury. Here, a feedback mechanism operates to reduce the translation of the arginase mRNA, which will give control over its copy number per cell, a situation that has yet to be modelled *in vitro*. Clearly, this does not happen when ornithine is generated freely outside the cell.

Pegylation definitely assisted in prolonging the half-life of arginase at 37°C by at least a factor of considerably more than two, although accurate assessments have yet to be made with time that clearly extends to weeks at 37°C. It is noteworthy that arginine deiminase can similarly be pegylated (Galea *et al*, 1996), but unfortunately arginine decarboxylase cannot (Table 2). The purity of arginase is high, and since it is activated at 60°C, most other proteins come out of solution and any contaminating enzymes are more likely to be *in*activated. We have run many experiments with 5% dialysed serum in the medium, and have found that there is no need to give additional Mn^2+^ in order to improve catalytic activity, and most preparations have been lyophilised with manganous salts present (Roholt and Greenberg, 1956). The strong inhibition of tumour cells by arginase indicates that it should be a very effective antitumour agent for treating animals, provided this effect can be translated into an effective *in vivo* protocol, as would seem possible from the work of Storr and Burton (1974), who obtained considerable tumour reduction after a remarkably short treatment time (3 – 4 days).

In contrast, arginine decarboxylase produces agmatine (Galea *et al*, 1996), which has the advantage both *in vivo* and *in vitro* of not being recycled to arginine. The bovine enzyme was as effective as the human recombinant form, and as arginase a unit for unit basis, but the former's specific activity could be up to two, if not three, orders of magnitude less than that of human recombinant arginase (Table 2). Therefore, much more protein would have to be injected into animals to produce the same effect, which might create a problem if the preparation was significantly immunogenic. Our data indicate no obvious problem with agmatine toxicity in culture (Figure 2C), except when it exceeded millimolar levels in the absence of arginine, and therefore some doubt remains as to whether a build-up under such conditions is accompanied by toxic sequelae in the body. Agmatine had toxic effects similar to those on HeLa cells on other cell lines, but its mode of action is not fully understood, although because it interferes with imidazoline receptor binding, it might block calcium channels (Santos *et al*, 2001). Interestingly, it is less toxic than spermidine, according to Katsuta *et al*. (1975), which may explain why it is needed at millimolar levels in the absence of arginine to have any effect.

However, agmatine is a small molecule that might be quickly cleared. While in some respects arginine decarboxylase could be an attractive enzyme to use, agmatine is also known to inhibit nitric oxide synthase (Galea *et al*, 1996). Furthermore, this enzyme requires pyridoxal-5-phosphate as a cofactor, but in culture we have found good activity in media where no additional cofactor was supplied. The downside of this enzyme, however, is that it is inactive following pegylation (Table 2), which is probably because of too few lysine side chains being available for attachment, and the few that are pegylated alter its conformation too drastically to retain activity. On balance, therefore, arginase remains the better candidate for *in vivo* use (Wheatley *et al*, 2002; Wheatley, 1998).

But there is another enzyme with some current popularity that we have not ourselves had available to compare throughout these studies, viz arginine deiminase. However, this third enzyme, prepared from cultures of *Mycoplasma arginini*, needs careful consideration before it can be considered of any value in tumour therapy because of some of the complications it produces. The enzyme's ability to degrade arginine explains why some mycoplasma infections can have devastating effects on cell growth (Terayama *et al*, 1982), but this is not confined to tumour cells. Unfortunately, a strict comparison of its catabolic activity relative to arginase and arginine decarboxylase has not yet been carried out in this regard, but we hope to report on this later. It is also claimed to be more effective than L-asparaginase against leukaemic cells *in vitro* (Gong *et al*, 2000), but this report took no account of their relative specific activities (Wheatley *et al*, 2002). It also apparently killed cells more effectively in arginine-containing medium than in AFM, which is incompatible with its primary action being through arginine deprivation. The product of arginine deiminase is citrulline, which will be quickly converted back to arginine both *in vivo* and *in vitro*, which further weakens this explanation, just as it does in other similar claims (Miyazaki *et al*, 1990; Sugimura *et al*, 1992; Takaku *et al*, 1992,^1993^). Therefore, the only truly rational explanation remaining is that the reaction generates ammonia. In cultures, where ammonia cannot be detoxified, small traces of the free gas can be lethal to both normal and tumour cells. The irony is that arginine itself is required to assist in the detoxification of ammonia. We do note, however, that some cells are incapable of utilising citrulline instead of arginine (Campbell *et al*, in press), and these might prove to be more susceptible to arginine deiminase provided it can be effective when arginine concentration is not too high (so that ammonia generation is limited).

### Complications with nitric oxide (NO) metabolism 

One further enzyme might also be further mentioned, viz nitric oxide synthase (Wu and Morris, 1998). This produces citrulline and NO from arginine, but this arginine has to be channelled initially from citrulline in what amounts to a tight circular coupling (Chang *et al*, 2001). It is of little use, therefore, as a catabolic enzyme, especially with an extremely low *K*_m_ (Table 1 and Table 2; Tatoyan and Giulivi, 1998), and its ability to deal with only nanomolar quantities of arginine shows it to be both inadequate and inappropriate. One apparent problem with arginine-catabolising enzymes such as arginase is that removal of arginine from the body was originally thought to deprive NO synthase of its substrate, and lead to loss of vascular tone and signalling in many other vital functions. However, new evidence strongly suggests that a special arginine pool may be generated, which is closely linked or channelled with nitric oxide synthase in appropriate cells (Flam *et al*, 2001).

### Implications for future applications

We still face problems with tumours and their hosts that are good converters of citrulline to arginine, although we have also clearly shown here (and the work in progress) that citrulline conversion cannot so often keep up with arginine demands of the faster-growing tumour cells. The most suitable subjects for treatment both in culture and *in vivo* will undoubtedly be those tumours that are poor converters, for example, certain hepatomas and melanomas (Sugimura *et al*, 1992).

A further problem that cannot be ignored in this complicated scenario is the control of the breakdown of nonessential proteins by the cells of the body (Grisolía and Wheatley, 1984), which can release amino acids in sufficient quantity to circumvent the best efforts to induce a true arginine-deficient state. We also do not know how much this, and scavenging from dead cells, plays a part in keeping at least some cells alive in arginine deprivation conditions after many days (Figure 5B). Some procedures can be introduced to help reduce protein breakdown, and these might be needed alongside the catabolic enzymes, although if the findings of Takaku *et al* (1993) are correct, pegylated arginine deiminase can keep arginine below detectable levels for at least 8 days in mice injected i.v. with a single bolus of 5 units of enzyme. Thus, it is difficult to assess the extent to which enzyme-induced deprivation will be affected by protein turnover without many more careful studies. Nevertheless, some cells can and do survive enzyme treatment or AFM in culture, even if these are very exceptional cases, after a period of a week or more of exposure. We are convinced that the only cells that seem to persist for any length of time are the very large multinucleated and often highly polyploid cells, as seen in HeLa and L1210 cultures (Figure 3). If Erenpreisa and Cragg (2001) are correct, then these large cells, similar to those surviving apparently lethal doses of radiation, may later undergo ‘reduction divisions’, creating one or two small progenitor cells that repopulate the cultures. However, it will take time to determine whether this is indeed the mechanism that also occurs in our cultures after arginine deprivation.

## CONCLUSION

We conclude that enzymatic degradation is a reasonable procedure for removing arginine from culture media, and this has also been shown to be effective *in vivo* by others, especially in pegylated forms of arginine deiminase and arginase. However, each enzyme has its shortcomings, and additional strategies will be needed to circumvent them. Arginase remains a strong contender, however, because of its high specific activity, and production of ornthinine rather than citrulline (Wheatley *et al*, 2002). The major obstacles are, (i) efficient recycling where an intact urea cycle operates, leaving only some of those in which the urea cycle has been compromised as susceptible, and (ii) in the large-scale protein turnover that can easily subvert efforts to maintain free arginine at micromolar levels.
